# Comparison of the severity of psychological distress among four groups of an Iranian population regarding COVID-19 pandemic

**DOI:** 10.1186/s12888-020-02804-9

**Published:** 2020-08-08

**Authors:** Amir Vahedian-Azimi, Malihe Sadat Moayed, Farshid Rahimibashar, Sajad Shojaei, Sara Ashtari, Mohamad Amin Pourhoseingholi

**Affiliations:** 1 Trauma Research Center, Nursing Faculty, Baqiyatallah University of Medical Sciences, Tehran, Iran; 2 Anesthesia and Critical Care Department, Hamadan University of Medical Sciences, Hamadan, Iran; 3 Basic and Molecular Epidemiology of Gastrointestinal Disorders Research Center, Research Institute for Gastroenterology and Liver Diseases, Shahid Beheshti University of Medical Sciences, Tehran, Iran; 4 Gastroenterology and Liver Diseases Research Center, Research Institute for Gastroenterology and Liver Diseases, Shahid Beheshti University of Medical Sciences, Tehran, Iran

**Keywords:** Coronavirus disease 2019, Psychological distress, Stress, Anxiety, Depression, Iran

## Abstract

**Background:**

Coronavirus disease 2019 (COVID-19) pandemic has caused serious psychological problems, including panic attack, anxiety, stress, and depression. The main objective of this study was to measure the prevalence and compare the severity of this psychological distress among four groups of an Iranian population.

**Method:**

In a cross-sectional survey, the mental health status of four groups of an Iranian society including community population, patients with COVID-19, medical staff, and medical students were investigated by the self-report questionnaire of Depression, Anxiety, and Stress Scale (DASS). DASS-21 questionnaire and the demographic data sheet were filled out by the participants. All statistical analyses were done using R version 3.6.1 software. *P*-values less than 0.05 were considered as statistically significant. ANOVA test was used to compare the severity of stress, anxiety, and depression between the four study groups.

**Results:**

Of the 886 participants in this survey, 554 (62.5%) were men and 332 (37.5%) were women, and the mean ± standard division of age was 40.91 ± 10.7 years. Among these participants, 241 (27.2%) were selected from community population, 221 (24.9%) were patients with COVID-19, 217 (24.5%) were medical staff, and 207 (23.4%) were medical students. The mean score of stress, anxiety, and depression in medical students and patients with COVID-19 was significantly higher than in medical staff and community population (*P* < 0.05). Overall, the anxiety score in men was higher than that in women (27.4 ± 4.6 vs. 26.48 ± 4.8, *P* = 0.006), and unmarried participants had a significantly higher depression score compared with the married group (27.5 ± 4.8 vs. 26.7 ± 4.6, *P* = 0.023). In addition, the score of depression was higher in female medical staff (27.08 ± 4.6 vs. 25.33 ± 4.3, *P* = 0.011) and community population (26.6 ± 4.3 vs. 25.3 ± 4.3, *P* = 0.02) than in male.

**Conclusion:**

COVID-19 patients and medical students in contact with these patients were at a high risk for mental illness due to lower experience compared with professional medical staff and community population. Continuous surveillance and monitoring of psychological distress for outbreaks should become a routine part of preparedness efforts worldwide.

## Background

The novel coronavirus disease 2019 (COVID-19), formerly known as severe acute respiratory syndrome coronavirus 2 (SARS-CoV-2), was first detected in December 2019 in Wuhan City in China [1]. As of 4 April 2020, more than 1,250,000 cases of COVID-19 were reported by more than 209 countries, hence characterized as a pandemic [2]. As a result of the rapid increase in the number of confirmed cases and deaths, both medical staff and the public have been experiencing psychological pressure and other health-related issues [3]. These concerns arose with all infections, including influenza, severe acute respiratory syndrome (SARS), and Middle-East respiratory syndrome (MERS) that took place years ago. During the outbreak of these infections, several psychiatric comorbidities such as depression, panic attack, anxiety, psychomotor excitement, suicide, and stress symptoms were reported [4–6]. However, COVID-19 has been highlighted as a unique threat that has added to panic, stress, anxiety, and the potential for depression due to its fast transmission pattern, inadequate preparedness of health officials, and the absence of a comprehensive and definitive treatment protocol or vaccination program [7].

Pandemics can psychologically affect everyone in the community. However, healthcare workers and medical students, particularly those involved in the treatment of COVID-19 patients, are highly susceptible to both infection and mental health problems. During previous SARS outbreak, research on healthcare workers showed that many presented high levels of psychological distress and frequent concerns regarding their health and functional ability and their families’ health [8–13]. Previous studies also revealed psychological disorders and chronic fatigue in the survivors of diseases such as SARS and MERS [14, 15]. Moreover, depression and post-traumatic stress disorders (PTSD) have been reported to last for as long as a year after illness [16]. There is a paucity of research on the psychological impact and mental health of the Iranian population during the COVID-19 pandemic. Therefore, we conducted this cross-sectional survey for the first time to measure the prevalence and compare the severity of the psychological distress (stress, anxiety, and depression) among community population, patients with COVID-19, medical staff, and medical students in an Iranian population. We hope our study findings will provide data support for the target intervention on psychological health in Iran and different parts of the world during the pandemic.

## Methods

### Study design

This cross-sectional survey was carried out from February to March 2020 in Tehran, Iran; the aim was to measure the prevalence and compare the severity of psychological distress (stress, anxiety, and depression) among community population, patients with COVID-19, medical staff, and medical students in an Iranian population. The present study was approved by the Ethics Committee of Baqiyatallah University of Medical Sciences, Tehran, Iran, with code IR.BMSU.REC.1398.442.

### Sample size

Cochran’s sample size estimation formula was used in the epidemiologic study. The first and second type errors were considered 0.05 and 0.02, respectively. A 50% satisfaction probability was assumed to estimate the maximum sample size for each group (196 participants). Given the nature of the study and the probability of dropout, a 10% drop was considered, and the final sample size was estimated to include a minimum of 216 participants in each group.

### Participants

Of the 886 participants in this cross-sectional survey, 241 (27.2%) were selected from community population, 221 (24.9%) were patients with COVID-19, 217 (24.5%) were medical staff, and 207 (23.4%) were medical students. Adult subjects aged 18 years or older, interested in participation, able to read and write, and having no physical disability or mental disorder (based on self-reports) were included in the community population group. Patients with COVID-19 were selected from those referred to Baqiyatallah Hospital, one of the main referral centers for specialized diagnosis and treatment of COVID-19, between February and March 2020 in Tehran, Iran. All patients with COVID-19 enrolled in this study were diagnosed according to World Health Organization interim guidance [17]. We included the medical staff members of Baqiyatallah Hospital who treated patients with COVID-19 infection for at least a week in February and March 2020 and volunteered to participate. Medical students included in the study were randomly selected from the faculty of nursing and medicine (Baqiyatallah University of Medical Sciences, Tehran, Iran). All the medical students in the study were interns working under the supervision of residents and fully licensed staff physicians at university Hospitals. Therefore, the included medical students had at least a week experience of working with COVID-19 patients.

### Data collection

DASS-21 questionnaire and the demographic data sheet were filled out by all participants. Demographic variables included age, gender, and marital status. In addition, work experience (years) and experience working with COVID-19 patients (weeks) were recorded for medical staff and medical students. The study participants were all informed about the objectives of the study and written informed consent was received from each participant. They were also assured of confidentiality.

### DASS-21 questionnaire

Mental health status was measured using the Depression, Anxiety, and Stress Scale (DASS-21). This questionnaire was designed and validated by Lovibond in 1995 [18] to measure the psychological distress in a community with 21 items. DASS-21 is a unique, simple, and approved instrument for assessing depression, anxiety, and stress both in clinical settings and communities [19]. DASS is a short screening tool that measures depression, anxiety, and stress by a 21-item self-report questionnaire. For each disorder, seven questions are considered, and the final score is obtained by the total score of the questions related to it. Each question was scored using a Likert-scale ranging from 0 (did not apply to me at all/never) to 3 (applied to me very much, or most of the time/almost always). Higher scores indicated a higher level of disorder based on a specific classification scoring system. Individuals were categorized into normal, mild, moderate, severe, and extremely severe based on their responses. Comparison of DASS-21 results with psychiatric interviews showed that this tool had a sensitivity and specificity of 75 and 89% and was capable of accurately screening depression, anxiety, and stress [20, 21]. The reliability and validity of the translated version of the Persian questionnaire was confirmed for an Iranian population. In a study by Sahebi et al. [22] on 970 students and military men, the translated questionnaire was reported to be comparable with the original one with a high internal correlation; Cronbach’s alpha of depression, anxiety, and stress subscales were 0.77, 0.79, and 0.78, respectively. In addition, the study by Moradipanah et al. [23] in Iran reported a Cronbach’s alpha of 0.94 for depression, 0.92 for anxiety, and 0.82 for stress.

### Statistical analysis

Categorical variables were described as frequency rates and percentages, and continuous variables were described using mean ± standard deviation (SD) values. The scores of the DASS subscales for each group were expressed as mean and standard deviation. ANOVA test was used to compare the severity of stress, anxiety, and depression between the four study groups. Moreover, the mean scores of stress, anxiety, and depression were compared between the two groups via (Tukey) post hoc test. All tests were two-tailed with a significance level of *P* < 0.05. Statistical analysis was performed using R version 3.6.1 software.

## Results

### Survey respondents

A total of 886 participants responded to the questionnaire. The results showed that the majority of participants had extremely severe anxiety 862/886 (97.3%), and the mean score of anxiety level was higher in men than in women (27.4 ± 4.6 vs. 26.48 ± 4.8, *P* = 0.006, 95% CI: 0.27–1.56). Meanwhile, in terms of stress (*P* = 0.446) and depression (*P* = 0.774), there was no statistically significant difference between men and women. In addition, unmarried participants had a significantly higher average depression score compared with the married group (27.5 ± 4.8 vs. 26.7 ± 4.6, *P* = 0.023, 95% CI: 0.11–1.38). However, the mean score of stress and anxiety levels did not differ between married and unmarried participants (*P* > 0.05).

### Demographic and psychological distress levels in the four study groups

Among these participants, 241 (27.2%) were selected from community population, 217 (24.5%) belonged to the medical staff, 207 (23.4%) were medical students, and 221 (24.9%) were patients with COVID-19. Table 1 shows the demographic characteristics and severity of psychological distress in the participants of the four study groups. The occupational status for community population and patients with COVID-19 was divided into five subgroups: employed, self-employed, looking for work or retired, student, and homemaker. The highest frequency in both groups was related to self-employed subgroup (more than 35%). However, this factor did not affect the level of stress, anxiety, and depression in these two groups.

**Table 1:** Demographic characteristics and severity of psychological distress of participants in four groups of study

Variables	Community population (*n* = 241)	Patients with COVID-19 (*n* = 221)	Medical staff (*n* = 217)	Medical students (*n* = 207)	Total (*n* = 886)
Age					
Mean ± SD (Range)	49.16 ± 8.01 (37–74)	45.90 ± 7.77 (33–71)	39.57 ± 6.71 (28–62)	27.37 ± 3.92 (20–38)	40.91 ± 10.7 (20–74)
Gender (%)					
Male	111 (51.2)	143 (69.1)	111 (51.2)	143 (69.1)	554 (62.5)
Female	106 (48.8)	64 (30.9)	106 (48.8)	64 (30.9)	332 (37.5)
Marital status (%)					
Married	151 (62.7)	99 (44.8)	158 (72.8)	120 (58)	528 (59.6%)
unmarried	90 (37.3)	122 (55.2)	59 (27.2)	87 (42)	358 (40.4%)
Stress (%)					
Mild	5 (2.1)	1 (0.5)	19 (8.8)	1 (0.5)	26 (2.9)
Moderate	106 (44)	94 (42.5)	103 (47.5)	63 (30.4)	366 (41.3)
Severe	118 (49)	103 (46.6)	84 (38.7)	128 (60.9)	431 (48.6)
Extremely severe	12 (5)	23 (10.4)	11 (5.1)	17 (8.2)	63 (7.1)
Anxiety (%)					
Moderate	0	0	1 (0.5)	0	1 (0.1)
Severe	10 (4.1)	6 (2.7)	5 (2.3)	2 (1)	23 (2.6)
Extremely severe	231 (95.9)	215 (97.3)	211 (97.2)	205 (99)	862 (97.3)
Depression (%)					
Moderate	25 (10.4)	16 (7.2)	32 (14.7)	3 (1.4)	78 (8.6)
Severe	124 (51.5)	85 (38.5)	101 (46.5)	60 (29)	370 (41.8)
Extremely severe	92 (38.2)	120 (54.3)	84 (38.7)	144 (69.6)	440 (49.7)

ANOVA test was used to compare the severity of stress, anxiety, and depression between the four groups under study (Table 2) including sex, marital status and age group (lower or upper 40 years); the results showed significant differences among four groups of study mean score of stress (F: 13.9, *P* < 0.001), anxiety (F: 14.8, *P* < 0.001), and depression (F: 23.9, *P* < 0.001) in. In addition, the mean scores of stress, anxiety, and depression were compared between the groups two by two with (Tukey) post hoc test. Results showed that the mean scores of stress, anxiety, and depression in medical students and patients with COVID-19 were significantly higher than the medical staff and community population (*P* < 0.05). Moreover, medical students had a significantly higher average depression score compared with COVID-19 patients (29.36 ± 4.4 vs. 28.07 ± 5.06, *P* = 0.031, 95% CI: 0.08–2.5). In terms of stress and anxiety, on the other hand, no statistically significant differences were detected between medical students and COVID-19 patients (*P* > 0.05).

**Table 2:** Comparison of anxiety, stress and depression scores based on DASS-21 questioner between 4 groups of study

Variables	Community population (***n*** = 241)	Patients with COVID-19 (***n*** = 221)	Medical staff (***n*** = 217)	Medical students (***n*** = 207)	***P***-value
**Stress**					
Mean ± SD (Range)	27.34 ± 4.37 (18–38)	28.59 ± 5.18 (18–44)	26.23 ± 5.62 (14–42)	28.99 ± 4.53 (18–40)	< 0.001*
**Anxiety**					
Mean ± SD (Range)	26.04 ± 4.52 (16–38)	27.62 ± 5.12 (16–42)	26.15 ± 4.24 (14–40)	28.56 ± 4.67 (16–42)	< 0.001*
**Depression**					
Mean ± SD (Range)	26.09 ± 4.39 (16–40)	28.07 ± 5.06 (16–46)	26.18 ± 5.09 (14–42)	29.36 ± 4.42 (18–42)	< 0.001*

* *P* < 0.05 was considered statistically significant. In addition, the mean score of stress, anxiety and depression were compared between the groups two by two in which (Tukey) post hoc test

According to the main effect model analysis, the depression score was significantly different between the groups according to gender (*P* = 0.015). The results showed that the average depression score in female medical staff (F/M: 27.08 ± 4.6 vs. 25.33 ± 4.3, *P* = 0.011, 95% CI: 0.39–3.08) and female community population (F/M: 26.6 ± 4.3 vs. 25.3 ± 4.3, *P* = 0.02, 95% CI: 0.21–2.47) was higher than in males. As far as age groups and marital status are concerned, no significant differences were observed between the groups according to stress, anxiety, and depression levels.

## Discussion

To our knowledge, this is the first study to report the prevalence and compare the severity of the stress, anxiety and depression among four groups of Iranian society at the initial stage of the COVID-19 outbreak. The main results of the present study showed that COVID-19 patients and medical students with at least 1 week experience of working with them had significantly higher scores of stress, anxiety, and depression compared to the medical staff and community population; this suggests that they are the main targets of psychiatric assessment and care. In total, the anxiety score was higher in men than in women, and unmarried participants had a significantly higher depression score compared with the married group. In addition, the score of depression in female medical staff and community population was higher in comparison with the males.

Previous studies have revealed an association between epidemics and mental disorders during the spread of infections such as SARS and MERS infection [24, 25]. Hawryluck et al. [4] reported that isolation and quarantine during the outbreak were stressful; they observed that certain subjects, due to quarantine for SARS in Canada, displayed symptoms of PTSD (28.9%) and depression (31.2%). Al-Najjar et al. [26] examined the psychological reactions of adults to the MERS epidemic in western Saudi Arabia; they found that anxiety was significantly increased by the increasing susceptibility to infection and social behaviors associated with travel and being in public places. Lee et al. [27] assessed the psychological impacts of the MERS outbreak and found that PTSD symptoms were very high among hospital staff even many years after the outbreak.

There are not many studies on the psychological impacts of COVID-19 pandemic on society, except for a few investigations conducted in China [28–30]. In a cross-sectional study by Wang et al. [31], psychological impacts, depression, stress, and anxiety were evaluated in 1210 participants from 194 cities in China at the beginning of the COVID-19 outbreak; their results showed that 53.8% of these individuals experienced severe psychological impacts of the outbreak. Moreover, 16.5, 28.8, and 8.1% of the respondents reported moderate to severe levels of depression, anxiety, and stress, respectively. In a cross-sectional observational study, Xiao et al. [32] measured the levels of anxiety, self-efficacy, stress, sleep quality, and social support in 180 medical staff; their findings showed that medical staff in China who were treating patients with COVID-19 infection had high levels of anxiety, stress, and self-efficacy that were dependent on sleep quality and social support.

The results of the present study and all previous studies show that the psychological trauma caused by the prevalence of infectious diseases is highly common in societies. Infectious pandemics can cause disruptions in societies and individuals on many levels [33]. COVID-19 imposes irreversible psychological impacts on all groups of community members, such as general population, healthcare workers, and students due to the commuting restrictions, fear of contracting the virus, anxiety about the closure of schools and businesses, the depression following the loss of friends and family, and fear of death [34–36]. Furthermore, according to the results of our study, the score of anxiety level in men was significantly higher than that in women. This could be attributed to the economic pressure caused by the pandemic because in most Iranian families, men are responsible for daily expenses. The economic impact of COVID-19 and its effects on community behavior, such as hoarding and stockpiling of resources and financial predicaments can cause psychological problems for the householder. Furthermore, the mean depression score in female medical staff and the female in the community population was higher than in men. In line with previous studies from China [31, 37], women seemed to experience elevated psychological symptoms related to this pandemic as compared to men. In this exceptional situation, women are faced with additional responsibilities, such as family care and child support in learning due to school closures. Female medical staffs are more likely to suffer from depression due to loneliness and separation from families and children. Hence, supporting women in this situation might be especially important.

The results of this study revealed that patients with COVID-19 and medical students in contact with these patients are the main targets of psychiatric assessment and care. Perhaps the main reason for the higher psychological distress among medical students compared with fully licensed physicians as medical staff is the former group’s lack of experience in controlling infectious diseases in high-risk environments and the fear of medical errors in the face of new cases. Other reasons are physical and emotional exhaustion due to a high-pressure health care system, rapid changes in medical information and procedures, self-perception of risk to themselves, the impact of pandemic on lifestyle, fear of inadequate medical equipment, such as masks and gloves, long working hours, and separation from families. To reduce the psychological distress among medical students, health policymakers are to introduce new policies that attract more medical students to the healthcare system with more awareness and readiness and also increase the flexibility of supervisors. Health services that employ interns must continue to supervise them and provide them, as much as possible, with meaningful educational clinical experiences. These interns should also be trained in caring forCOVID-19 patients, informed about protective measures, and supported during challenging times.

Unpredictability, uncertainty, seriousness of the disease, misinformation, social isolation, and the overwhelming news may cause anxiety and fear in the public. The general public may also experience boredom, frustration, and irritability under isolation measures [29]. In patients with COVID-19, these can be related to the fear of severe disease consequences, contagion, isolation treatment, loss of trust in health services, and fear of death. Consequently, they may experience loneliness, denial, anxiety, depression, insomnia, and despair, which may lower the treatment adherence. Some of these cases may even run increased risks of aggression and suicide. Due to the sudden shock of the outbreak and the lack of information on the disease, interventions to promote mental well-being may not be possible at the beginning of global pandemics. As a possible solution for this challenge, public health decision makers need to perform appropriate psychosocial interventions and incorporate mental health management plans in next few months. Other steps to lowering the psychological distress in society can be the assessment of the accuracy of information, enhancing social support, reducing the stigma associated with the disease, maintaining a normal life while adhering to safety measures, and using available psychosocial services.

The main strengths of the present investigation were the comparison of the severity of this psychological distress among the four groups of society and the large sample size in each group. However, this study is not without its limitations. First, this is a single-center cross-sectional survey which limits the generalizability of our findings. Our participants in three groups of study were recruited in the same hospital, which cannot represent the Iranian population, hence the need for further research. Second, we were unable to investigate the history of participants’ mental disorders. Thus, participants with a history of mental disorders based on self-report were excluded from the study.

## Conclusion

This study showed the high severity of anxiety, stress, and depression among Iranian subpopulations during COVID-19 pandemic. COVID-19 patients and the medical students in contact with them were the main targets of psychiatric assessment and care. Increasing public awareness, building trust in the media, and providing information on patients’ recovery can reduce the psychological burden of this pandemic in the society. In addition, based on the previous experiences with SARS outbreak, some patients and health professionals are traumatized by the COVID-2019 outbreak and still suffer from persistent psychiatric symptoms even after the outbreak. Therefore, we should expect long-term negative psychological outcomes as post-traumatic stress disorder (PTSD) among COVID-19 survivors and healthcare workers. This indicates the necessity of designing psychological interventions so as to improve mental health during and after the pandemic.

### Ethics approval and consent to participation

The present study was approved by the Ethics Committee of Baqiyatallah University of Medical Sciences, Tehran, Iran, with code IR.BMSU.REC.1398.442. The study participants were all informed about the objectives of the study and written informed consent was received from each participant.

## Data Availability

All data collected and analyzed during the current study can be provided by the corresponding author on reasonable request.

## Abbreviations

COVID-19: Coronavirus Disease 2019
DASS: Depression, Anxiety and Stress Scale
PTSD: Post-traumatic stress disorders
SARS: Severe acute respiratory syndrome
MERS: Middle-East respiratory syndrome
